# Workload and use factor data for a modern digital radiography system

**DOI:** 10.1002/acm2.13962

**Published:** 2023-03-21

**Authors:** Krystal M. Kirby, Beth A. Schueler, Laurel A. Littrell, Zaiyang Long

**Affiliations:** ^1^ Department of Radiology Mayo Clinic Rochester Minnesota USA

**Keywords:** digital radiography, pediatric radiography, shielding, workload

## Abstract

The well‐referenced structural shielding design NCRP Report No. 147 uses workload information based on self‐reported film‐screen data from the AAPM Task Group 9 survey. The aim of this study was to assess the clinical workload distributions of modern digital radiography (DR) systems in general hospital and pediatric‐only practices. A retrospective analysis of DR imaging data on four radiographic systems in a hospital practice and two radiographic systems in a pediatric practice, through a custom clinical DICOM header analytics program. A total of 203, 294 exposures from the general hospital practice and 25,415 from the pediatric practice from 2019 and 2021 were included. Values for kVp, mAs, and detector type (wall bucky, table bucky, or free detector) were extracted. For each exam, mAs was accumulated in a kVp histogram with bins 5 kVp wide and further parsed by detector type. Total workload was calculated by summing all exposures, then normalized by the number of patients. The median (25th and 75th percentile) workload in the hospital practice was 0.43 (0.22, 1.13) mA‐min per patient, while the average was 1.36 ± 3.08. Pediatric data yielded a median (25th and 75th percentile) of 0.10 (0.05, 0.23) and an average of 0.29 ± 0.69 mA‐min per patient. Mean number of patients per week was 230 adult and 57 pediatric. Hospital workload data is approximately 44% less than the NCRP Report No. 147 value.

## 1 INTRODUCTION

In radiography practices, radiation shielding designs for X‐ray imaging machines are performed prior to equipment installation. To calculate the shielding requirements for a diagnostic imaging room, the shielding designer must determine not only the usage and distribution of the X‐ray tube, but also the number of patients per week, room dimensions, and occupancies of adjacent areas.

The current gold standard for radiation shielding designs for diagnostic X‐ray facilities is the NCRP Report Number 147^1^ (NCRP 147), though other reports were used before 2005.^2^ In this report, workload is the metric used to quantify the output of the X‐ray tube. The workload is defined as the time integral of the X‐ray tube current over a specified period, usually expressed in milliampere‐minutes (mA‐min) and also normalized over the number of patients. Using the AAPM Diagnostic X‐ray Imaging Committee Task Group 9^3^ (AAPM TG 9) survey of 735 patients with 2479 film‐screen exposures in radiographic rooms across seven institutions, the workload data between the chest bucky wall and floor (or other barriers) were separately accumulated to reflect the different exam types and kV distributions. Therefore, in NCRP 147, shielding a general radiography room requires use of two different workloads: chest bucky and floor/other barriers. In addition to the workload, the use factor, *U*, is defined as the fraction of the workload directed toward a given primary barrier.

Digital radiography (DR) has been broadly implemented in the past decade. It utilizes flat panel digital detectors, which have a higher detective quantum efficiency than film‐screen,^4^, ^5^ theoretically allowing for a lower radiation output to achieve the same desired image quality. However, the larger dynamic range of DR can lead to an increase in the exposure beyond the level required to get adequate or superior image quality, the latter of which is known as dose creep. Therefore, even though a higher patient throughput is expected, the effect of an increased dynamic range is not as straightforward. In addition, potential changes in patient body habitus and imaging tasks could also play a role on the workload data. Overall, the effects from switching to DR on workloads, primary beam use factors, and patient numbers have not yet been evaluated.

For shielding pediatric DR rooms, AAPM TG9 survey included patients of all ages and did not report pediatric specific data. In reality, pediatric imaging techniques differ from those for adults. Pediatric patients are on average smaller in thickness than the general population, which theoretically would result in a lower workload for the same number of patients. For pediatric imaging of body parts with water‐equivalent thicknesses of less than certain threshold, such as 10−12 cm, the use of antiscatter grids is also not recommended.^6^, ^7^ The omission of grids in pediatric patients can decrease dose without a substantial decrease in image quality.^8^ The types of exams performed may also differ. Some exams may be performed more frequently in pediatric populations, such as evaluations of skeletal development.

The aim of this study was to present the results of a survey for modern DR equipment in both general hospital and pediatric practices. Workload distributions were calculated per wall and parsed by kVp, and use factors were determined for each practice type.

## 2 METHODS

A retrospective survey of general diagnostic DR exams performed in calendar years 2019 and 2021 was conducted. Surveyed units in a general hospital practice (24 h, 7d) included two each of these scanners: Discovery XR656 (GE Healthcare, Milwaukee, WI) and Ysio Max (Siemens Healthineers, Erlangen, Germany). The year 2020 was excluded since the COVID‐19 pandemic significantly affected the radiography workloads. All diagnostic radiographic exposures were included, though the Ysio Max machines were installed in 2020 and here only included data from 2021. In addition, two Ysio (Siemens Healthineers, Erlangen, Germany) systems in a pediatric‐only practice (8 h, 5 d) were also investigated.

A Digital Imaging and Communications in Medicine (DICOM) receiver was set up to receive clinical images from all above‐mentioned scanners at the general hospital and pediatric practices, including DICOM for‐presentation images as well as for‐processing and rejected images whenever possible. A custom DICOM analytics program was written in MATLAB (MathWorks, Natick, MA) to extract and store both public and private DICOM metadata.^9^ Relevant DICOM tags (Table 1) were extracted, such as study date, presentation intent type, station name, peak kilovoltage (kVp), and milliampere‐seconds (mAs). Station name was used to ensure inclusion of only the relevant DR scanners. Presentation intent type was used to differentiate for‐processing and for‐presentation images on GE systems, and only exposures tagged for‐processing were included. Since one exposure can yield multiple processed images, this ensures each exposure is counted only once and will exclude stitched images. For exposures taken on the Ysio Max and Ysio systems, the study description was preceded by a letter identifying the type of detector used (T = table bucky, W = wall bucky, X = free detector). For exposures on GE systems, the detector type was identified through the private DICOM tag 0011−1044. Accession numbers were used to calculate the total number of patients. This study obtained exempt review approval by the local Institutional Review Board.

**TABLE 1:** List of relevant DICOM tags and attribute descriptions.

Tag	Attribute description
0008‐0020	Study date
0008‐0068	Presentation intent type
0008‐1010	Station name
0008‐103E	Series description
0008‐0050	Accession number
0018‐1020	Peak kilovoltage (kVp)
0018‐1152	Exposure in mAs
0017‐10CA	Binary for image rejection (Siemens only)
0011‐1044	Type of detector (GE only)

Repeat‐reject analysis was performed by analyzing year 2021 for rejected images (Siemens machines only). General hospital and pediatric practices were analyzed separately. For the general hospital practice where rejected DICOM images were harvested, each clinical image that was marked as a rejected image by its reject DICOM tag (0017, 10CA) was used in calculations for a repeat‐reject rate (RRR) per body part (18 total). Specifically, the RRR was determined by dividing the number of rejections by the number of exposures for that body part. For pediatric data, rejected images were pulled from the quality assurance tool located on each scanner. Since the pediatric data included a lower number of exams, the overall RRR for 2021 was used to correct the data, without separation by body part.

After RRR correction, the data was reduced for each practice type and barrier type (floor, cross‐table wall, or chest bucky wall) by accumulating the workload in mAs for each exposure in its corresponding 5‐kVp wide bin, creating a workload spectrum. The normalized workload spectrum was generated by dividing the total workload in each kVp bin by the total number of patients per spectrum. Exams included in the cross table wall were axillary shoulder, lateral hip, and decubitus chest, as well as some trauma exposures of the lateral cervical, thoracic, and lumbar spine. It is difficult to definitively summarize the exams directed toward the chest wall bucky and floor, due to individual projection requirement, patient status, and technologist selection. Common exam types for the floor could include upper extremities, abdomen, pelvis, femur, tibia/fibula, as well as some projections of the knees, ankles and feet; and for the chest wall bucky, skull, spine, chest, scoliosis, hip‐to‐ankle, and other weight‐bearing exams.

Use factors were determined by calculating the fraction of the total workload for which the primary beam was directed at that specific barrier.

## 3 RESULTS

**FIGURE 1:**
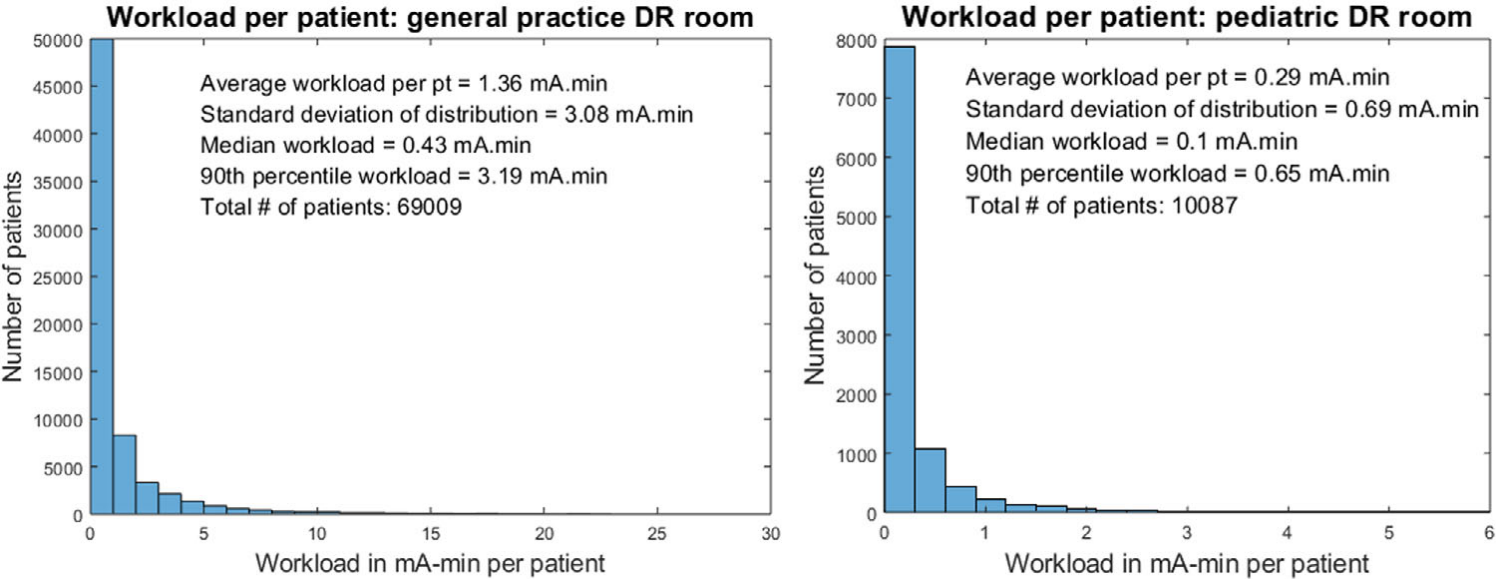
Distribution of workload per patient in mA‐min for each of the general DR and pediatric practices.

Workload data for each calendar year is shown in Table 2 and Figure 1. The median (25th and 75th percentile) workload for the general hospital practice was 0.43 mA‐min (0.22, 1.13) per patient, with mean and standard deviation of 1.36 ± 3.08 mA‐min per patient. The 90th percentile is also reported. For the pediatric practice, the median (25th and 75th percentile) workload was 0.10 (0.05, 0.23) mA‐min per patient, with mean and standard deviation 0.29 ± 0.69 mA‐min per patient. The average number of patients was 230 per week in the general hospital practice and 57 per week in the pediatric practice, per room surveyed.

**TABLE 2:** Mean and median workload in mA‐min per patient for general hospital and pediatric practices.

	General practice	Pediatric practice
Mean workload ± standard deviation	1.36 ± 3.08	0.29 ± 0.69
Median workload (25th, 75th percentile)	0.43 (0.22, 1.13)	0.10 (0.05, 0.23)
90th percentile	3.19	0.65
# Exposures	203294	25415

**FIGURE 2:**
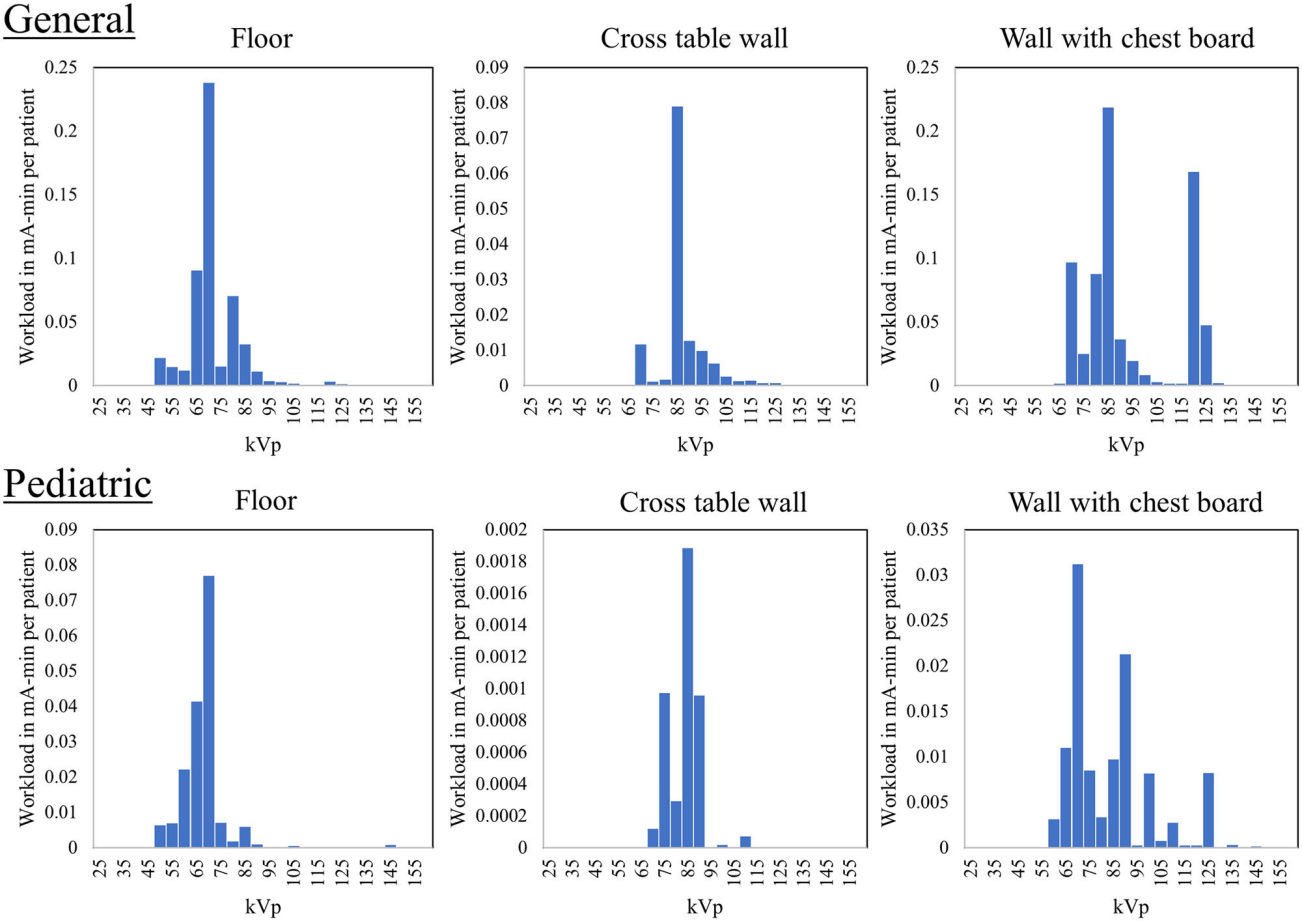
Workload distributions by kVp bin, graphically representing the data in Table 4.

Table 3 shows the workload distributions in 5‐kVp‐wide bins over a range of kVp values from 25 kVp to 160 kVp, and a graphical representation is shown in Figure 2. Note that this is the average workload per barrier type, not components of the overall workload. The floor, cross table wall, and wall with the chest bucky are included for each of the general and pediatric practices.

**TABLE 3:** Workload spectrum in mA‐min, normalized per patient. The kVp in each row refers to the highest value in each bin.

	General practice DR room				Pediatric DR room		
kVp	Floor	Cross table wall	Wall with chest board	kVp	Floor	Cross table wall	Wall with chest board
25	0	0	0	25	0	0	0
30	0	0	0	30	0	0	0
35	0	0	0	35	0	0	0
40	0	0	0	40	0	0	0
45	4.59E‐06	0	0	45	1.65E‐06	0	0
50	2.18E‐02	0	0	50	6.41E‐03	0	0
55	1.47E‐02	1.43E‐06	4.72E‐05	55	6.96E‐03	0	9.91E‐06
60	1.18E‐02	2.99E‐05	1.71E‐04	60	2.22E‐02	0	3.13E‐03
65	9.05E‐02	7.78E‐05	1.47E‐03	65	4.14E‐02	0	1.10E‐02
70	2.38E‐01	1.17E‐02	9.69E‐02	70	7.69E‐02	1.19E‐04	3.12E‐02
75	1.50E‐02	1.11E‐03	2.50E‐02	75	7.04E‐03	9.73E‐04	8.49E‐03
80	7.03 E‐02	1.73E‐03	8.75E‐02	80	1.80E‐03	2.94E‐04	3.33E‐03
85	3.24E‐02	7.90E‐02	2.19E‐01	85	5.85E‐03	1.88E‐03	9.72E‐03
90	1.11E‐02	1.26E‐02	3.65E‐02	90	9.98E‐04	9.58E‐04	2.13E‐02
95	3.64E‐03	9.86E‐03	1.95E‐02	95	1.32E‐05	0	2.28E‐04
100	2.88E‐03	6.24E‐03	8.36E‐03	100	1.98E‐04	1.65E‐05	8.17E‐03
105	1.37E‐03	2.54E‐03	2.90E‐03	105	5.77E‐04	0	7.37E‐04
110	8.41E‐04	1.33E‐03	1.65E‐03	110	9.91E‐05	7.27E‐05	2.73E‐03
115	1.98E‐04	1.48E‐03	1.49E‐03	115	3.30E‐06	0	2.36E‐04
120	3.28E‐03	6.91E‐04	1.68E‐01	120	7.44E‐05	0	2.61E‐04
125	1.04E‐03	7.01E‐04	4.72E‐02	125	0	0	8.23E‐03
130	4.51E‐05	2.35E‐04	2.07E‐03	130	0	0	4.96E‐06
135	1.29E‐05	1.66E‐04	8.08E‐04	135	0	0	3.04E‐04
140	2.17E‐05	1.43E‐04	5.14E‐04	140	0	0	0
145	8.74E‐06	0	1.07E‐04	145	7.90E‐04	0	1.39E‐04
150	0	8.60E‐05	3.39E‐04	150	1.16E‐05	0	1.65E‐06
155	0	0	0	155	0	0	0
160	0	0	0	160	0	0	0
W_total_	0.52	0.13	0.72		0.17	0.004	0.11

Use factors are shown in Table 4. There was a change in use factors from 2019 to 2021 in the general practice, but not in the pediatric practice

**TABLE 4:** Use factors for general and pediatric practices.

	General practice		Pediatric	
	2019	2021	2019	2021
Floor	0.74	0.55	0.69	0.69
Cross table wall	0.04	0.03	0.03	0.03
Chest bucky wall	0.22	0.42	0.28	0.28

Overall RRR for general hospital and pediatric practices were 11.0% and 6.9%, respectively. The body part rejection percentages are shown in Table 5. The highest RRR was the pelvis with a 22.8% RRR and the lowest RRR was 2.5% for vertebral columns (for scoliosis imaging).

**TABLE 5:** List of the body part tags and their corresponding percentage of rejected images for the general hospital practice in 2021.

Body part tag	Rejection %
‘ABDOMEN’	7.1
‘ANKLE’	13.9
‘CHEST’	8.4
‘CLAVICLE’	11.4
‘CSPINE’	21.7
‘ELBOW’	13.9
‘EXTREMITY’	10.1
‘FOOT’	10.2
‘HAND’	5.2
‘HIP’	17.5
‘KNEE’	17.5
‘LSPINE’	19.1
‘PELVIS’	22.8
‘SHOULDER’	17.2
‘SKULL’	18.2
‘SSPINE’	9.5
‘TSPINE’	13.7
‘VERTEBRAL COLUMN’	2.5

## 4 DISCUSSION

The switch from film‐screen to DR represents a shift in technique factors in modern radiography practice, and new shielding surveys are needed to evaluate this change. For example, workload surveys including digital breast tomosynthesis units yielded significantly higher workload values than reported in NCRP 147 for mammography.^10^, ^11^ This current study investigated modern DR workload data and use factors in a general hospital practice and a pediatric practice, using direct DICOM header analytics with a large sample size of images within one institution. Due to the non‐normal distribution of workload data, we report the median value for the workload in addition to the mean, standard deviation, and 90th percentiles, while AAPM TG 9 report did not include the median value except for one unit. To make a direct comparison, the mean workload in this survey for the general hospital practice (1.36 mA‐min per patient) yielded a percent difference of 44% lower than the AAPM TG 9 reported value (2.45 mA‐min per patient). The median is 0.43 mA‐min per patient in this study, in contrast to the 1.17 mA‐min per patient from one unit in the TG 9 survey. Pediatric data showed a much lower mean and median (0.29 and 0.10 mA‐min per patient, respectively) workload per patient than the general practice, as expected due to the lower average patient thickness and higher utilization of non‐grid projections. Though the majority of workload per patient values were tightly distributed for both general and pediatric practices indicated by the 90^th^ percentile values, the overall widespread data from various exam and patient types yielded standard deviations that were on the order of or higher than the means, which was similar to the TG 9 survey.

The workload spectrum showed drastically different kVp distributions for each wall type. In the general practice, the majority of the workload directed at the floor occurred in the range between 65 and 85 kVp, since it consisted of many shoulder, extremity, abdomen, pelvis, femur, and tibia/fibula exams. This kVp pattern is similar in the pediatric population, as the kVp values for these floor pediatric exams are the same or near the same range with adults, although with a much lower workload. The cross table wall exhibits a high peak at 85 kVp, indicative of two types of exams that are typically performed at that kVp (axillary shoulder and lateral hip exams) for common patient thicknesses. The only wall for which there was a significant number of high‐kVp exams (>100 kVp) was the wall with the chest bucky. Compared to NCRP 147, the kVp distributions are very similar in shape, with the main difference being the workloads at 120 kVp. Since most chest imaging is done at this kVp, the presence of dedicated chest radiography rooms in the NCRP 147 data and the lack of these rooms in our data was the most likely cause of this disparity. Furthermore, some exams utilize compensating filters mounted on the X‐ray tube, such as the C‐ and T‐spine filters, the lateral hip filter, as well as the Thoraeus filter for pediatric airway imaging. The use of these external compensating filters could have a slight impact on the current workload data.

A 2013 study from Taiwan^12^ found an average total workload of 0.42 mA‐min per patient, lower than the data reported here for two main reasons. First, the average number of exposures per patient exam can differ from country to country. The average number of exposures per patient was 3.0 in this report, compared to an average of 1.6 in Taiwan. Second, the average patient size in Taiwan is smaller than that in the U.S.^13^ However, the workload distribution spectrum over kVp is very similar, since the distribution shape is determined primarily by the modality type. Minor differences are due to differences in exposure techniques as well as specific examination frequencies.

In the current study, the use factors for the general practice changed comparing 2019 to 2021, most likely due to a change in our practice. In early 2020, dedicated chest rooms were replaced by new scanners with general purpose. Therefore, the current use factors for 2021 may be more similar to other institutions where dedicated chest rooms are not used. The COVID‐19 pandemic may also have an impact, although our practice data showed significantly more portable chest X‐ray exams which are not relevant for this study. Furthermore, the presentation of this data differs from that of NCRP 147 since we directly report use factors. Though the normalized workload per wall already takes wall usage into account, the use factors are reported separately to illustrate and compare trends in room usage between years. Calculating radiation shielding requirements using this report requires knowledge of the installing site's own use factors and patient numbers. And generally, a conservative approach would be beneficial to account for uncertainties and future changes.

A comparison between patient numbers in this report and TG 9 is not direct, since this report included a 24 h/7 d hospital practice while the TG 9 report was for an 8 h/5 d practice. The main reason for using a 24 h/7 d hospital practice was to include a variety of exams types to give average workload values that are more generalizable to other practices. Many 8 h/5 d DR rooms at our institution are specialized (for example, for orthopedic practice), or smaller satellite sites with lower patient volume. Therefore, the difference between the 230 patients per week in this report and the 120 (average) and 160 (busy) in TG 9 is due to both the extended hours of the general hospital practice (accounting for about 40% of patients) and the implementation of DR in place of screen‐film. Typically, the most straightforward number to calculate is the average number of current patients, with consideration for future growth.

Additionally, this is the first report of pediatric‐only DR workload and use factors. With comparable use factors to the general practice, it is reasonable to conclude that the decrease in workload would result in lower shielding requirements. This is especially relevant in a pediatric‐only practice or hospital since those rooms would likely only image pediatric patients in the future. However, if it is a pediatric‐only room in a general practice, conservative design would be wise in case if the room may be changed to image other patients in the future.

Even though repeat‐reject analysis was not the main goal of this study, it showed an overall 11% RRR for the hospital practice, which is comparable to literature results for DR.^14^, ^15^, ^16^ The reject reasons were technologist self‐reported, mainly including patient positioning, image artifacts, and underexposure. Patient positioning for exposures that utilize AEC can be challenging especially for projections such as the lateral lumbar spine, as proper alignment of the AEC cells under the correct body part is not visually apparent, and thus these exposures contributed to more rejected images. Image quality may also be more critical for hospital patients. Special exams could also be reflected by the RRR data. For example, the vertebral column/scoliosis exams are taken by a small, focused group of technologists for an orthopedic requirement and are typically not repeated.

There are a number of limitations. First, the current study only included a single large institution, and technique factors and use patterns may differ slightly in other practices. Second, the study utilizes clinical DICOM header information and is retrospective in nature. Rejected DICOM image information are not necessarily always available. Therefore, RRR was computed separately for the two types of practice for correction of workload data. Last but not least, primary and secondary air kerma measurements were not made, but will be studied in the near future.

## 5 CONCLUSION

Significant workload data difference has been shown for modern DR rooms in general hospital and pediatric practices, compared to literature film‐screen data.

## AUTHOR CONTRIBUTIONS

Krystal M. Kirby, Beth A. Schueler, and Zaiyang Long were involved in the study conceptualization and design. Zaiyang Long was responsible for data collection. Krystal M. Kirby performed data analysis and manuscript drafting. All were involved in data interpretation and manuscript review. Laurel A. Littrell supported and provided clinical expertise and feedback to this project. All authors approved the final manuscript.

## CONFLICT OF INTEREST STATEMENT

No conflicts of interest.
